# ERK signaling waves via body-wall muscles guide planarian whole-body regeneration across long distances

**DOI:** 10.1186/s13619-023-00180-9

**Published:** 2023-11-08

**Authors:** Chenglu Xiao, Jing-Wei Xiong

**Affiliations:** 1 National Key Laboratory of Veterinary Public Health and Safety, College of Veterinary Medicine, China Agricultural University, Beijing, 100193 China; 2 College of Future Technology, Peking University, Beijing, 100871 China; 3 College of Basic Medical Sciences, The Second Affiliated Hospital, Nanchang University, Nanchang, 330031 China

## Abstract

Whole-body regeneration is a multifaceted process that reinstates a body to its initial three-dimension size and structure after resection injury. It is well-known that signaling waves such as calcium and extracellular signal-related kinase (ERK) signaling waves can efficiently transmit information between tissues or cells. However, the mechanisms responsible for coordinating wound responses over long distances are largely unexplored. A recent study has reported that the propagation of ERK signaling waves *via* longitudinal body-wall muscles play an essential role in wound response and whole-body regeneration in planarians, underscoring the significance of feedback interactions between spatially distinct tissues during whole-body regeneration over long distances. These findings not only address the central questions of regenerative biology but also have potential implications for regenerative medicine.

## Main text

The comprehensive coordination of cellular dynamics at the tissue level that is necessary for appropriate morphogenesis and regeneration, encounters difficulties due to the substantial dimensions of regenerating body parts (Bertero and Murry 2018; Foex 1999). It has been widely reported that injury can elicit reactions in unaffected tissues located at a considerable distance from the site of the wound, implying that signals from wounds can travel over great distances (Johnson et al. 2018; Rodgers et al. 2014; Sun et al. 2022). However, we have limited knowledge about the mechanisms that can timely transmit molecular signals over distances ranging from millimeters to centimeters among different tissues. A recent article highlights that injury-triggered extracellular signal-related kinase (ERK) signaling waves *via* longitudinal body-wall muscles guide planarian whole-body regeneration over long distances (Fan et al. 2023). In this short article, we briefly review several recent studies on the discovery of signaling waves, with a particular focus on long-distance ERK signaling waves, during regeneration.

In order for spatiotemporal coordination to occur, regenerative signals need to propagate through both space and time. Ways in which signaling molecules can spread through space are well recognized as diffusion and active transport (Deneke and Di Talia 2018; Hasegawa et al. 2015). However, the speed of diffusion is insufficient to synchronize biological events that take place over long distances within seconds to minutes, and active transport requires specialized molecular motors (Deneke and Di Talia 2018). One way to overcome these limitations is through the use of traveling chemical waves, which can spread a signal both in space and time (Gelens et al. 2014; Ishihara et al. 2014). Besides the classic Ca^2+^ waves and transmission of action potentials along neuronal pathways, other signaling waves such as Cdk1, Thrombin, and Notch waves have been shown to have significant impact on a variety of biological processes (Dashkevich et al. 2012; Deneke et al. 2016; Jiang et al. 2000). Importantly, Hiratsuka and colleagues found that the epidermis displays bursts of ERK activation patterns after injury, where the ERK signal spreads from one cell to another in a radial wave by using a transgenic fluorescent ERK reporter; and ERK activation propagates in parallel with the wound edge (Hiratsuka et al. 2015). Furthermore, the ERK signaling waves are associated with the cell-cycle, providing novel perspectives on the coordination of cell proliferation and transient ERK activity within a living tissue (Hiratsuka et al. 2015).

Another elegant work presents the critical role of rhythmic travelling EKR waves in regulating the tempo-spatial growth of bone in regenerating zebrafish scales (De Simone et al. 2021). By creating a transgenic ERK kinase translocation reporter (KTR) strain, the authors performed real-time live imaging of the ERK activity, quantitative analysis, and mathematical modeling, and revealed that the activation of ERK signaling occurs in a repetitive pattern, with waves that move across the population of osteoblasts in a concentric manner (5 or 6 ERK waves in each scale during the entire period of longitudinal regeneration). The ERK waves are slowed by Mek inhibitor treatment, and small ERK waves are slower than larger ones, suggesting that ERK activity spreads as trigger waves. When ERK activity was blocked with the MEK inhibitor PD0325901, the tissue flows and expansion of tissue rings were greatly disrupted during regeneration. Thus, the trigger ERK waves serve as an effective regulatory strategy to coordinate cell behavior and guide tissue formation during the process of regeneration (De Simone et al. 2021).

When examining the process of regeneration in vertebrates, our attention is typically directed towards a single organ, such as limb, brain and heart, etc. Regeneration of these organs usually takes weeks to months and requires the activation of a number of specific cell pools. And identifying remote responses and mapping communication networks between organs pose significant technical challenges. Planarian flatworms have the ability to regenerate into fully functioning organisms from small pieces of tissues in just a few days. Injury causes extensive alterations in gene expression across the entire organism. The ability to regenerate rapidly together with the rapid response of whole-body tissues at the molecular level makes planarians a valuable model for studying the response of distal tissues to injury and indicates the presence of injury signal that can move at a high speed. The ERK signaling waves are proposed to engage in long-distance communication, but their reported velocity, ranging from 10 to 100 μm/h, is insufficient to account for the initiation of distant wound reactions in planarians (De Simone et al. 2021; Deneke and Di Talia 2018; Fan et al. 2023; Hiratsuka et al. 2015). It remains unexplored how the ERK waves propagate quickly over long distances. Interestingly, Fan and colleagues have recently reported that the Erk signaling waves disseminate at ∼1 mm/h and guide planarian regeneration, which is comparable to the speed of certain intracellular trigger waves in large cells. They observed the ERK activity peaks at ~ 0.5 h post amputation in tissues proximal to the wounds, and it spreads to distal areas with a delay of ~ 1 h in the peak time between neighboring positions. Inhibition of ERK propagation by using specific concentrations of Mek inhibitors results in failure of distal tissue regeneration that can be rescued by a second injury within certain time. Most interestingly, they hypothesized and formalized a propagation model to clarify how ERK activation can propagate so quickly. In this model, the longitudinal body-wall muscles, which are long and could form dense parallel tracks, act as superhighways for propagating the ERK signaling waves. And the morphological properties of muscle cells influence the propagation of ERK signals, the speed of signal propagation increases with the length of relay cells, long cell volume density and the orientation of long cells. The relay model also suggested when there are long cells, smaller cells may have a limited effect on the transmission of signals and become activated by signals from the long cells. And the main functions of distal response might be providing feedback to the proximal injured region (Fan et al. 2023). Thus, this work reveals, for the first time, that wound signals are able to propagate over distances of several millimeters within a few hours, and the distal wound feedback is important for whole-body regeneration in planarians. Although Fan et al. stated that the main conclusions in their model may be generalizable to other complex tissue systems, cellular structure is known to determine function, and the unique muscle organization of planarians is important for the rapid propagation of ERK waves, leading to a rapid distal response to injury. Whether such a rapid distal response to vertebrate organ regeneration exists and whether this response is dependent on rapidly propagating ERK waves remains to be investigated.

## Conclusions

**Fig. 1:**
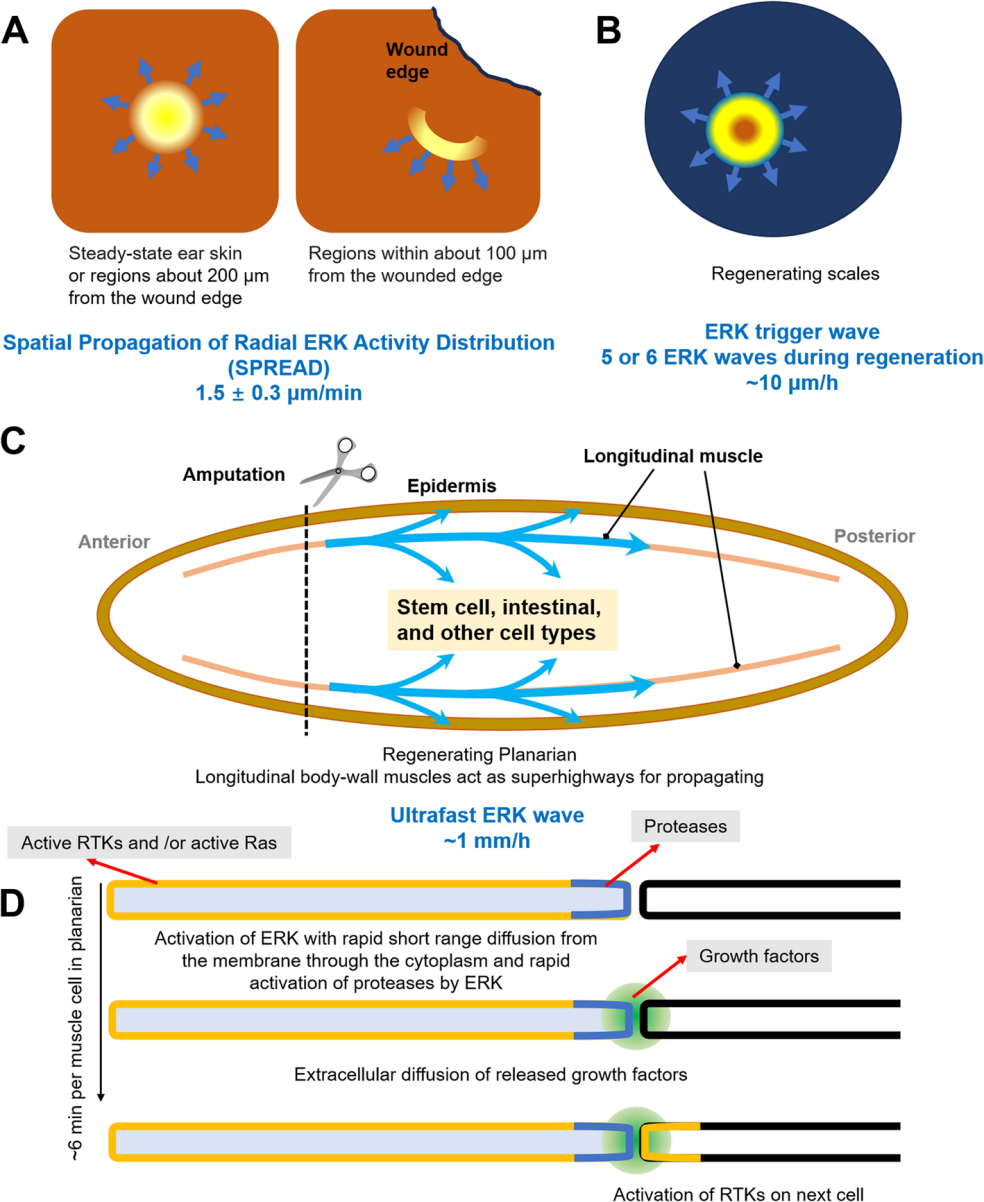
Three models on ERK signaling waves in different kinds of tissues.** A** Schematic of two-type ERK activity propagation patterns in the epidermis (Hiratsuka et al. 2015). **B** Schematic of ERK activity waves that travel across regenerating scales (De Simone et al. 2021). **C** Ultra-fast ERK waves during planarian whole-body regeneration (Fan et al. 2023). **D** Schematic of ERK activity propagation between long cells during planarian regeneration (Fan et al. 2023)

Together, these studies highlight the important phenomenon that local injury initiates and relays a complex signaling network that guides long-distance tissue regeneration, thus elucidating not only the mechanisms of transmission of damage signals over long distances, but also emphasizing the significance of distant tissue feedback to damage signals during regeneration (Fig. 1). Both mTORC1 and hepatocyte growth factor activator are necessary for satellite cells distant from the injury to enter an “alert” phase and thereby respond to injury (Rodgers et al. 2014, 2017). The *cebpd-linked enhancer* is essential for activating *cebpd* at a distance and regulating fluid levels following cardiac injury (Sun et al. 2022). The ERK signaling waves are found not only in tissues composed of uniform cell populations, but also in multiple cell-type tissues, and the injury signals can transmit from the wound site to distal tissues *via* ERK waves (De Simone et al. 2021; Fan et al. 2023; Hiratsuka et al. 2015). Further investigations are warranted to uncover the types of injury-trigger signals, additional cellular and molecular mechanisms of long-distance signal propagation, and the feedback mechanisms of distal tissues during organ and whole-body regeneration.

## Data Availability

Not applicable.
